# Microbiota-derived metabolites as drivers of gut–brain communication

**DOI:** 10.1080/19490976.2022.2102878

**Published:** 2022-07-28

**Authors:** Hany Ahmed, Quentin Leyrolle, Ville Koistinen, Olli Kärkkäinen, Sophie Layé, Nathalie Delzenne, Kati Hanhineva

**Affiliations:** a Food Sciences Unit, Department of Life Technologies, University of Turku, Turku, Finland; b Metabolism and Nutrition Research Group, Louvain Drug Research Institute, UCLouvain, Brussels, Belgium; c School of Medicine, Institute of Public Health and Clinical Nutrition, University of Eastern Finland, Kuopio, Finland; d School of Pharmacy, University of Eastern Finland, Kuopio, Finland; e Laboratoire NutriNeuro, UMR INRAE 1286, Bordeaux INP, Université de Bordeaux, Bordeaux, France; f Department of Biology and Biological Engineering, Division of Food and Nutrition Science, Chalmers University of Technology, Gothenburg, Sweden

**Keywords:** Gut microbiota, gut-brain axis, metabolism, metabolites, short-chain fatty acids

## Abstract

Alterations in the gut microbiota composition have been associated with a range of neurodevelopmental, neurodegenerative, and neuropsychiatric disorders. The gut microbes transform and metabolize dietary- and host-derived molecules generating a diverse group of metabolites with local and systemic effects. The bi-directional communication between brain and the microbes residing in the gut, the so-called gut–brain axis, consists of a network of immunological, neuronal, and endocrine signaling pathways. Although the full variety of mechanisms of the gut–brain crosstalk is yet to be established, the existing data demonstrates that a single metabolite or its derivatives are likely among the key inductors within the gut–brain axis communication. However, more research is needed to understand the molecular mechanisms underlying how gut microbiota associated metabolites alter brain functions, and to examine if different interventional approaches targeting the gut microbiota could be used in prevention and treatment of neurological disorders, as reviewed herein.

**Abbreviations:**4-EPS 4-ethylphenylsulfate; 5-AVA(B) 5-aminovaleric acid (betaine); Aβ Amyloid beta protein; AhR Aryl hydrocarbon receptor; ASD Autism spectrum disorder; BBB Blood–brain barrier; BDNF Brain-derived neurotrophic factor; CNS Central nervous system; GABA ɣ-aminobutyric acid; GF Germ-free; MIA Maternal immune activation; SCFA Short-chain fatty acid; 3M-4-TMAB 3-methyl-4-(trimethylammonio)butanoate; 4-TMAP 4-(trimethylammonio)pentanoate; TMA(O) Trimethylamine(-*N*-oxide); TUDCA Tauroursodeoxycholic acid; ZO Zonula occludens proteins

## Introduction

The circulating metabolome is influenced by factors including nutrition, health status, and the gut microbiota composition.^1^ Further, it is possible to predict the blood metabolome through the collection of nutritional, clinical, and microbial data highlighting the host-gut microbiota crosstalk.^1–3^ As the tight relationship between gut microbiota composition and circulating metabolome has become evident, it is essential to focus on the concomitant evaluation of nutritional intake and the clinical profile alongside microbiota composition.

The central nervous system (CNS) and the intestine form a multifaceted, bidirectional communication network where signals are conveyed by nervous, endocrine, and immune systems. In this so-called ‘gut–brain axis’, the gut microbiota signals the brain through the mentioned networks by activating sympathetic^4^ and parasympathetic^5^ neurons in the intestine, by educating the immune system^6^ and by regulating the production of several neurotransmitters^7–9^ and gut hormones.^10–13^ Furthermore, bacterial metabolites are of special interest as they include known neuromodulators,^14,15^ uremic toxins,^16^ pro-inflammatory^17^ and anti-inflammatory mediators^18,19^ and molecules providing energy for host’s cellular metabolism.^20,21^ Some metabolites of interest have been implicated in brain functions, such as neurodevelopment and regulation of neuroinflammation, as well as blood–brain barrier (BBB) integrity.

**Figure 1.**
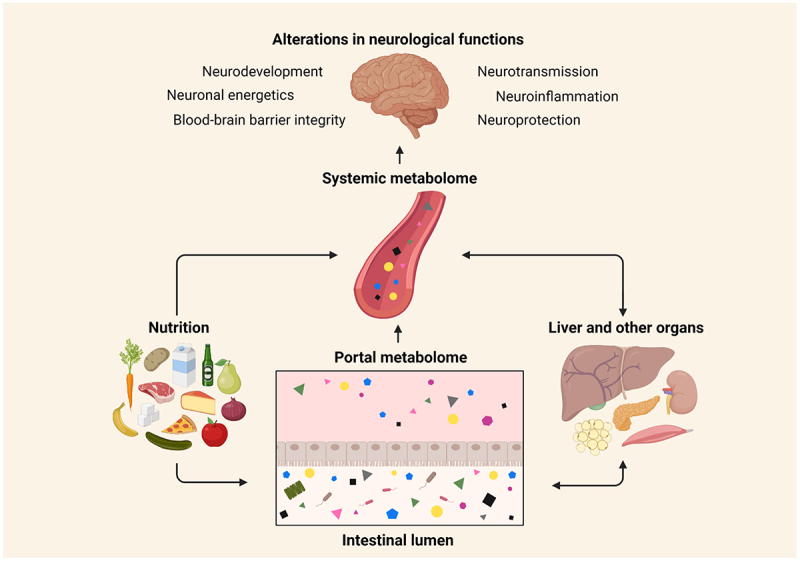
Host metabolic homeostasis and neurological impact.

The analysis of the circulating metabolome reflects the crosstalk between nutrition, microbiome, and host metabolism (summarized in Figure 1). Therefore, it may reveal potential biomarker candidates of health and disease, enable understanding of related metabolic processes and can be helpful in rationalizing individual responses to preventive or therapeutic interventions (Box 1). In addition, modulating certain type of neuroactive metabolites, by acting on nutrition and gut microbiota, represents an interesting strategy to prevent and treat neurologic and neuropsychiatric diseases. In this review, our focus is on the small metabolites with a mass below 1600 Da and we describe how neuroactive metabolites produced by the gut microbiota from dietary source are linked to key features of neuronal processes and dysfunction and risk of health outcomes thereafter (Table 1).

The portal metabolome reflects the intestinal metabolism of dietary compounds. After processing by the liver, a pool of metabolites will reach the systemic circulation. The systemic metabolome is a dynamic metabolic signature reflecting the interaction between the diet, gut microbiota, liver, and other organs. Metabolites can influence the functioning of all organs, including the brain by modulating a range of metabolic pathways related to neurological functions. (Figure created with Biorender.com)

Box 1. **Gut-derived metabolites: Biomarker or effector? Friend or foe?**

**Table:** 

Some gut-derived metabolites such as trimethylamine-*N*-oxide (TMAO), indoles and phenylacetylglutamine have been strongly associated with pathological outcomes including cardiovascular diseases and death. Robust studies showed that even after adjusting for several confounding factors (i.e. other cardiovascular risk factors) these metabolites can predict death from cardiovascular diseases.^22–24^ However, there are conflicting observations as phenylacetylglutamine has been highlighted as a biomarker of healthy aging being associated with a shift in microbiome composition toward increased uniqueness, a marker of good health in aged subjects.^25^ It confirmed previous data showing elevated levels of this metabolite in centernarians.^26^ Phenylacetylglutamine has also been associated with an increased α-diversity and abundance of ‘beneficial’ bacteria (*Akkermansia*, genus from *Christensenellaceae*) despite its potential detrimental effects on cardiovascular health.^24^ Regarding TMAO, a recent work highlighted that trimethylamine (TMA) is detrimental for the BBB integrity while TMAO was protective and promoted cognitive performance in mice.^27^ Several other preclinical studies showed that TMAO can be beneficial against atherosclerosis, nonalcoholic fatty liver disease and glucose homeostasis impairments and is required for neurodevelopment.^28–31^ Indole derivatives, despite their associations with cardiovascular diseases, have been shown to exert anti-inflammatory effects on liver and to be beneficial in inflammatory bowel disease or experimental autoimmune encephalomyelitis model of multiple sclerosis.^32–35^ The levels of these metabolites are influenced by several factors such as dietary intake of nutrients (e.g., choline, phenylalanine and tryptophan), gut microbiota composition and function, elimination including metabolization (e.g., in the liver) and renal excretion. Interestingly, studies found that TMAO levels are greatly influenced by the presence of a type-2 diabetes or a kidney disease and that elevation of this compound can be due to confounding factor or a reverse causation.^36^ Fish consumption, recognized for its health-promoting effect, also leads to elevated levels of circulating TMAO.^37^ Better understanding of the origin of metabolites and their altered levels due to dietary intake, gut microbiota production or host metabolism will help to clarify the relationship between metabolome and health on individual level. Studying the compounds’ effect, alone or in combination, at physiological dose is also important to distinguish what happens in pathological conditions (e.g., loss of renal or hepatic function) or in basal condition. Thus, future studies should gather nutritional, metabolomic, microbial and clinical data to be able to control for confounding factors.

**Table 1.** Gut microbiota-associated metabolites, their biological pathway, preclinical setups, and main outcomes and associations to clinical studies. Direction of outcomes are indicated by symbols as followed; increase (↑) and decrease (↓).

Mechanism of action	Common name	Chemical formula	Origin	Preclinical model	Main preclinical outcomes	Clinical association	Reference
**Neurodevelopment**							
	3-(3-sulfooxylphenyl) propionic acid Indoxyl sulfate Phenyl sulfate Pyrocatechol sulfate	C_9_H_10_O_6_S C_8_H_7_NO_4_S C_6_H_5_O_4_S C_6_H_6_O_5_S	Xenobiotic metabolism Tryptophan metabolism Xenobiotic metabolism	SPF, GF and gnotobiotic C57BL/6 J mice	Fear extinction learning ↑ Altered learning-related neuronal activity	n.a	^38^
	4-EPS	C_8_H_10_O_4_S	Tyrosine metabolism	C57BL/6 N MIA mice SPF and GF C57BL/6 J mice *Ex vivo* brain tissue cultures	Anxiety-like behavior ↑ Oligodendrocyte maturation ↓ CNS neuronal myelination ↓ Altered brain activity patterns	Fecal levels in ASD children ↑	^39–41^
	5-AVAB Hippuric acid Imidazole propionic acid TMAO	C_5_H_11_NO_2_ C_9_H_9_NO_3_ C_6_H_8_N_2_O_3_ C_3_H_9_NO	Lysine metabolism Xenobiotic metabolism Choline and carnitine metabolism Histidine metabolism	*Ex vivo* C57Bl/6 J mice thalamic, striatal and hypothalamic explants of axons	Fetal thalamocortical axonogenesis ↑	n.a	^28^
	5-AVA Taurine	C_5_H_11_NO_2_ C_2_H_7_NO_3_S	Lysine metabolism Bile acid and cysteine metabolism	Humanized and GF C57BL/6 miceBTBR T^+^ tf/J mice	Repetitive behavior ↓ Social behavior ↑ Anxiety-like behavior ↓	n.a	^42^
	*p*-Cresol	C_7_H_8_O	Tyrosine and phenylalanine metabolism	NOD mice C57BL/6 mice Mouse primary oligodendrocyte cultures	Social behavior ↓ Medial prefrontal cortex myelination ↓	Fecal and urinary levels in ASD children ↑	^16,43,44^
	Acetic acid Propionic acid	C_2_H_4_O_2_ C_3_H_6_O_2_	Dietary fiber fermentation	Obese C57BL/6 J mice dams and offspring	Social behavior ↑ Memory scores↑ Microglia maturation ↑Improved synaptic ultrastructure	Altered fecal SCFA profiles in ASD children	^43,45–47^
	Indole	C_8_H_7_N	Tryptophan metabolism	GF, SPF and *E. coli* monocolonized C57BL/6 J mice *Ex vivo* neural progenitor and stem cell cultures	Neuronal maturation ↑ Neurogenesis ↑	n.a	^36^
**Neurotransmission**							
	4-aminobenzoic acid Deoxycholic acid Tyramine	C_7_H_7_NO_2_ C_24_H_40_O C_8_H_11_NO	Folate metabolism Bile acid metabolism Tyrosine metabolism	SPF, GF and monocolonized C57Bl/6 J, Slc6a4 KO, Swiss Webster and Rag1 KO mice RIN14B (ATCC) cell cultures	EECs stimulation ↑ Serotonin biosynthesis by EECs ↑	n.a	^9^
	*p*-Cresol	C_7_H_8_O	Tyrosine and phenylalanine metabolism	Chronic (p.o.) or acute (iv, ip) administration to Wistar rats and C57BL/6 J or BTBR T^+^ tf/J mice	Altered brain dopamine metabolism Anxiety-like behavior ↑Social behavior ↓	CSF levels ↑ in Alzheimer’s and Parkinson’s disease patients	^48–51^
	Butyric acid Isobutyric acid Isovaleric acid Norepinephrine	C_4_H_8_O_2_ C_4_H_8_O_2_ C_5_H_10_O_2_ C_8_H_11_NO_3_	Dietary fiber fermentation Tyrosine/phenylalanine metabolism	*Ex vivo* C57BL/6 J mice intestinal organoids and colonic neuron cultures HEK293T (ATCC) cell cultures	EECs are stimulated by microbial metabolites Serotonin biosynthesis by EECs ↑ EECs modulate primary afferent nerve fibers	n.a	^52^
	Dopamine Norepinephrine	C_8_H_11_NO_2_ C_8_H_11_NO_3_	Tyrosine and phenylalanine metabolism	GF and SPF BALB/c mice BALB/c mice monocolonized with *E. coli*	Gut microbes transform luminal dopamine and norepinephrine into biologically active form	n.a	^7^
	GABA	C_4_H_9_NO_2_	Glutamic acid metabolism	BALB/c mice treated with *L. rhamnosus* (JB-1) probiotics	Altered brain GABA and glutamate/glutamine levels Anxiety-like behavior ↓ Depression-like behavior ↓ Stress ↓	Altered GABA and glutamate in postmortem brains of persons with history of heavy alcohol use	^5,14,53,54^
	Histamine Phenethylamine	C_5_H_9_N_3_ C_8_H_11_N	Phenylalanine and histidine metabolism	C57Bl/6 mice monocolonized with *M. morganii* HEK293 cell cultures	*M. morganii* strains generate trace amines activating dopamine- and histamine-receptors	Microbial histidine decarboxylase genes are enriched in patients with Chron’s disease	^15^
	Indole	C_8_H_7_N	Tryptophan metabolism	F344 rats monocolonized with *E.coli* Acute (i.p.) administration to F344 rats Mouse EEC cultures	Acute GLP-1 secretion from EECs ↑ Anxiety-like behavior ↑ Helplessness ↑	n.a	^55,56^
	Indole Indole-3-aldehyde	C_8_H_7_N C_9_H_7_NO	Tryptophan metabolism	HEK-293 T mammalian cell cultures *Ex vivo* human and mouse small intestine tissue sections	Activation of EECs via TRP1 receptor ↑ Serotonin biosynthesis by EECs ↑	Indole derivatives were positively associated with memory scores	^57,58^
	Kynurenic acid	C_10_H_7_NO_3_	Tryptophan metabolism	Kynurenin aminotransferase II KO mice Acute (i.c.v.) administration to Sprague-Dawley or Long Evans rats	Brain glutamate levels ↑ by limiting brain kynurenic acid supply Learning ↓ Memory scores ↓	Decreased plasma tryptophan and kynurenic acid levels in patients with depressionCognitive functions negatively correlated to plasma kynurenine/tryptophan ratio	^59–64^
	Lactate	C_3_H_6_O_3_	Dietary fiber fermentation	Hooded rats treated with *Lactobacillus*- and *Bifidobacterium* probiotics C57BL/6NTac mice monocolonized with Lactobacillus strains	Plasma and brain lactate ↑ GABA expression and signaling ↑ Memory, learning and neuroplasticity ↑	n.a	^20,65,66^
	Pipecolic acid	C_6_H_11_NO_2_	Lysine metabolism	GF and SPF BALB/c mice	Levels of pipecolic acid in the cerebrum ↑ in the presence of microbiota	n.a	^67^
	Serotonin	C_10_H_12_N_2_O	Tryptophan metabolism	*Lactobacillus* and *Streptococcus* cultures	*In vitro* production of biogenic amines, including serotonin	Circulating levels decrease in substance use disorders	^68–70^
	Tryptamine	C_10_H_12_N_2_	Tryptophan metabolism	Humanized or *B. thetaiotaomicron* monocolonized Swiss Webster, 129/Sv and 5-HT4R KO mice Colonic organoids	Activation of colonic serotonin receptor 4 ↑ Colonic secretion ↑	n.a	^71^
**Modulation of BBB integrity**							
	*p*-Cresol glucuronide	C_13_H_16_O_7_	Tyrosine metabolism	Acute (i.p.) administration to C57BI/6 mice Human CMEC/D_3_ cultures	BBB permeability ↓ LPS-stimulated BBB permeability↓	n.a	^72^
	Acetic acid Propionic acid	C_2_H_4_O_2_ C_3_H_6_O_2_	Dietary fiber fermentation	C57BL/6 J mice monocolonized with *B. thetaiotaomicron*	BBB permeability ↓ Tight junction protein expression ↑	n.a	^73^
	Butyric acid	C_4_H_8_O_2_	Dietary fiber fermentation	C57BL/6 J mice monocolonized with *C. tyrobutyricum*GF and SPF C57BL/6 J, BALB/c and NMRI mice Acute (ip) administration to C57BL/6 TBI mice	BBB permeability ↓ Tight junction protein expression ↑ Histone acetylation ↑ Neurological deficits ↓	n.a	^73,74^
	Deoxycholic acid	C_24_H_40_O_4_	Bile acid metabolism	Acute (i.v.) administration to sham or BDL-operated Sprague Dawley rats Rat BMEC cultures	Impairments in tight junction integrity Phosporylation of occludin ↑	Serum levels associated with cognitive decline and cerebrospinal fluid t-tau aggregation in Alzheimer’s disease patients	^75–77^
	Propionic acid	C_3_H_6_O_2_	Dietary fiber fermentation	Human postmortem brain endothelium and CMEC/D_3_ cell cultures	Oxidative stress ↓ LPS-stimulated BBB permeability ↓	n.a	^78^
	TMA	C_3_H_9_N	Choline and carnitine metabolism	C57BL/6 J mice Human CMEC/D_3_ cultures	Impairments in barrier integrity Cytoskeleton disruption and metabolic stress ↑	n.a	^27^
	TMAO	C_3_H_9_NO	Choline and carnitine metabolism	Chronic (i.p.) and acute (i.p.) administration to C57Bl/6 J mice Human CMEC/D_3_ cultures	Improved barrier integrity Astrocyte and microglial function ↑	Cerebrospinal fluid levels ↑ in Alzheimer’s disease patients and associated with markers of Alzheimer’s disease	^27,79^
	Ursodeoxycholic acid	C_24_H_40_O_4_	Bile acid metabolism	Human BMEC cultures	Improved barrier integrity Endothelial cell apoptosis ↓	Serum and postmortem brain levels ↑ in Alzheimer’s disease patients	^76,80,81^
**Neuroinflammation**	Acetic acid	C_2_H_4_O_2_	Dietary fiber fermentation	Chronic (p.o.) administration to GF and SPF C57BL/6 or 5× FAD micePrimary glia cell cultures*Ex vivo* isolated mice microglia	Microglial metabolism ↑ Microglia maturation ↑ Aβ plaque deposition ↑ in SPF mice but not in GF mice	n/a	^82^
	Acetic acid Butyric acid Propionic acid	C_2_H_4_O_2_ C_4_H_8_O_2_ C_3_H_6_O_2_	Dietary fiber fermentation	Chronic (p.o.) administration to GF C57BL/6 mice Chronic (p.o.) administration to surgically or sham-treated Sprague-Dawley rat model of CCH Primary glia cell cultures*ex vivo* brain endothelial cell cultures Chronic (p.o.) administration to GF or SPF Thy1-αSyn and GF APPPS1 mice	Microglial activity ↑ Microglia maturation ↑ Neuroinflammation, cognitive decline and depressive-like behavior ↓ rat model of CCHα-syn aggregation ↑ in genetically predisposed SPF mice Aβ plaque deposition ↑ in genetically predisposed GF mice	Brain amyloid load positively associated with plasma acetic acid and inversely associated with plasma butyric acid in patients with cognitive complaints	^83–87^
	Dihydrocaffeic acid	C_9_H_10_O_4_	Xenobiotic metabolism	Chronic (p.o.) administration to CD45.2+ C57BL/6 miceMice PBMC and neuron tissue cultures	Pro-inflammatory cytokine production ↓	n.a	^88^
	Indoxyl sulfate	C_8_H_7_NO_4_S	Tryptophan metabolism	Acute (i.p.) administration to C57BL/6 mice Chronic (p.o.) administration to Albino Wistar rats *Ex vivo* rat and mice glial cell cultures	Oxidative stress ↑ Pro-inflammatory cytokine production ↑ Locomotor activity ↑ Spatial memory ↓ Apathic behavior ↑	CSF levels ↑ in patients with Parkinson’s diseaseUrinary levels positively associated to recurrent depressive symptoms	^17,51,89,90^
	Indole Indole-3-aldehyde Indolepropionic acid Indoxyl sulfate	C_8_H_7_NO C_9_H_7_NO C_11_H_11_NO_2_ C_8_H_7_NO_4_S	Tryptophan metabolism	C57BL/6 J EAE and CX3CR1-AHR mice *Ex vivo* C57BL/6 J mice microglia and astrocyte cell cultures Primary human microglia, brain and fetal astrocyte cultures	Type 1-interferon signaling ↓ EAE disease scores ↓ Pro-inflammatory cytokine expression ↓	Serum AhR activating tryptophan metabolites ↓ in patients with multiple sclerosis	^32,91^
	*N^6^*- carboxymethyllysine	C_8_H_16_N_2_O_4_	Lysine metabolism	Chronic (i.p. or p.o.) administration to SPF and GF C57BL/6 mice Human cortical tissue and mice brain preparations Bone marrow-derived macrophage cultures	Oxidative stress in microglia ↑ Microglial dysfunction ↑	Associated with oxidative stress in brain tissue of elderly and patients with Alzheimer’s disease and/or diabetes mellitus	^92–94^
	Propionic acid	C_3_H_6_O_2_	Dietary fiber fermentation	Acute (i.c.v or i.v.) administration to Western albino, Long-Evans, seizure-prone or seizure-resistant rats Chronic (p.o.) administration to C57BL/6 EAE mice*Ex vivo* mice Treg-cell cultures	Astrogliosis and microglial activity ↓ Hyperactivity ↑ Social behavior ↓ Inflammatory cytokines and oxidative stress ↑ Treg-cell differentiation ↑ CNS autoimmunity ↑	n.a	^95^^–100^
	TMAO	C_3_H_9_NO	Choline and carnitine metabolism	Chronic (p.o.) administration to surgically or sham treated F344× BN F1 rats	Hippocampus oxidative stress ↑ Neuroinflammation ↓ Postoperative cognitive decline ↑	Positive and inverse associations with Parkinson’s or Alzheimer’s diseases	^101–105^
	Urolithin A	C_13_H_8_O_4_	Xenobiotic metabolism	Chronic (p.o.) administration to C57BL/6 EAE mice*Ex vivo* C57BL/6 mice CNS mononuclear and dendritic cell cultures*Ex vivo* 2D2 TCR mice Th17 cell culturesMurine BV-2 microglia cell cultures SPF mice model of ischemic stroke	Pro-inflammatory cytokine gene expression ↓ Microglia, dendritic and T-cell activation ↓ Neuroinflammation ↓ Neuronal apoptosis ↓	n.a	^93,94,106–108^
**Neuronal energy metabolism**							
	3M-4-TMAB 4-TMAP	C_8_H_18_NO_2_C_8_H_18_NO_2_	Carnitine metabolism	GF and SPF C57BL/6 J mice Primary murine CNS white matter cell culture	Fatty acid oxidation in CNS white matter tissue ↓	n.a	^109^
	Acetic acid Butyric acid Indolepropionic acid Propionic acid TUDCA	C_2_H_4_O_2_ C_4_H_8_O_2_ C_11_H_11_NO_2_ C_3_H_6_O_2_ C_26_H_45_NO_6_S	Dietary fiber fermentation Tryptophan metabolism Dietary fiber fermentation Bile acid metabolism	Chronic (p.o. or i.p.) administration to *db/db* mice	Improved cognitive functionsImproved mitochondrial function Mitochondrial biogenesis and function ↑	n.a	^110^
	Butyric acid Propionic acid	C_4_H_8_O_2_ C_3_H_6_O_2_	Dietary fiber fermentation	Chronic (i.c.v.) administration to Long-Evans rats	Locomotor activity ↑ Altered brain fatty acid profiles	n.a	^111^
	Butyric acid	C_4_H_8_O_2_	Dietary fiber fermentation	Neuroblastoma or lymphoplastoid cell cultures derived from ASD males	Improved mitochondrial function Mitochondrial gene expression ↑	n.a	^112^
	Ethanol	C_2_H_5_OH	Energy metabolism	SPF or humanized C57BL/6 J mice	Depression-like behavior ↑ Circulating β-hydroxybutyrate ↓ Social behavior ↓	Microbial ethanol ↑ together with β-hydroxybutyrate ↓ in alcohol-dependent subject with behavioral alterations	^113^
	Lactate	C_3_H_6_O_3_	Energy metabolism	Chronic (p.o.) administration to CC042 miceC57BL/6NTac mice monocolonized with *Lactobacillus* species Exercised or acute (i.p.) administration to C57BL/6 mice	Long-term memory formation ↑ Hippocampus GABA ↑ Learning ↑ Hippocampus BDNF ↑	n.a	^65,66,114^
	Indolepropionic acid	C_11_H_11_NO_2_	Tryptophan metabolism	Mice neuro2a-APP_sw_ cell cultures	Restoration of cell respiratory rate Reactive oxygen species ↓	n.a	^115^
**Neuroprotection**							
	3-(3′-hydroxyphenyl) propionic acid 3-hydroxybenzoic acid	C_9_H_10_O_3_ C_7_H_6_O_3_	Xenobiotic metabolism	Chronic (p.o.) administration to Sprague-Dawley rats Synthetic Aβ preparations	Aβ plaque deposition ↑ Neuroplasticity ↑	n.a	^116^
	Ergothioneine	C_9_H_15_N_3_O_2_S	Histidine metabolism	Chronic (p.o.) administration to stressed Sprague Dawley rats or Aβ-injected C57BL/6 mice	Intracerebral oxidative stress ↓ Aβ plaque deposition ↓ Depression-like behavior ↓ Social behavior ↑	Plasma levels ↓ in patients with mild cognitive impairment or Parkinson’s disease	^117,118^
	Ferulic acid	C_10_H_10_O_4_	Xenobiotic metabolism	Rat pheochromocytoma cell culturesChronic (p.o.) administration corticosterone-treated Swiss mice, PSAPP mice or Sprague-Dawley rats with acute ischemia	Oxidative stress ↓ Aβ plaque deposition ↓ Neuronal apoptosis ↓ Depression-like behavior ↓	n.a	^18,119,120^
	Indolepropionic acid	C_11_H_11_NO_2_	Tryptophan metabolism	SK-N-SH human neuroblastoma cell cultures E-18 fetal rat primary neuron cell cultures	Neuronal apoptosis ↓ Oxidative stress ↓	n.a	^121^
	Menadione Menaquinone Phylloquinone	C_11_H_8_O_2_ C_31_H_40_O_2_ C_31_H_46_O_2_	Energy metabolism	α-Synuclein fibril preparations	α-syn aggregation ↓	n.a	^122^
	TUDCA	C_26_H_45_NO_6_S	Bile acid metabolism	Chronic (p.o.) administration to APPPS1 mice	Aβ plaque deposition ↓ Neuronal degeneration ↓	n.a	^123^

Abbreviations: 3 -M-4-TMAB, 3-methyl-4-(trimethylammonio)butanoate; 4-EPS, 4-ethylphenylsulfate;; 4-TMAP, 4-(trimethylammonio)pentanoate: 5-AVA(B), 5-aminovaleric acid (betaine); Aβ, amyloid beta protein; AD, Alzheimer’s disease; AhR, aryl hydrocarbon receptor; ASD, autism spectrum disorder; BDL, bile duct ligation; BMEC, brain microvascular endothelial cells; CCH, chronic cerebral hypoperfusion; CNS, central nervous system; EAE, experimental autoimmune encephalomyelitis; EEC, enteroendocrine cell; GABA, γ-aminobutyric acid; GF, germ-free; I.C.V, intracerebroventricular; I.P., intraperitoneal; I.V., intravenous; MIA, maternal immune activation; NOD, nonobese diabetic; PBMC, peripheral blood mononuclear cell; P.O., per os; SCFA, short-chain fatty acid; SPF, specific-pathogen free; TBI, traumatic brain injury; TMA(O), trimethylamine(-*N*-oxide); TUDCA, tauroursodeoxycholic acid.

## Microbiota-related metabolites modulating brain function

### Neurodevelopment

Mouse models devoid of gut microbiota, the germ-free (GF) mice, elicit characteristics supporting the view that the gut microbiota is crucial for the normal brain development and behavior.^124,125^ Desbonnet et al.^126^ showed that despite of normal gut microbiota at birth, mice treated with antibiotics in the early weeks of life display cognitive and behavioral alterations later in life. Given that the neurodevelopmental processes are initiated in the prenatal phases, maternal microbiota, and related molecules may influence fetal development as observed in the field of early immune development.^127–129^ The maternal immune activation (MIA) mice model of autism spectrum disorder (ASD) was used in the pivotal work by Hsiao et al.^39^ to study how the maternal microbiota influences offspring’s brain development. The postnatal serum metabolomic analyses revealed that the level of 4-ethylphenylsulfate (4-EPS), a tyrosine-derivative, was 46-fold higher in MIA offspring compared to control offspring. Postnatal treatment with the human commensal *Bacteroides fragilis* improved several features of ASD in MIA offspring and restored the 4-EPS levels which were explained by the improvements in intestinal permeability and thus decreased translocation of 4-EPS to the circulation. The microbiota modulates the production of 4-EPS as it was nearly undetectable in GF compared to specific pathogen-free mice. Additionally, administration of 4-EPS to naive wild-type mice at postnatal weeks 3–6 induced anxiety-like behavior similar to MIA offspring. The biosynthetic pathway and mechanisms behind the detrimental effects of 4-EPS were lately elucidated by Needham et al.^40^ showing that 4-EPS interfered with oligodendrocyte maturation, myelination, and brain activity patterns in brain areas of the limbic system. *p*-Cresol, a tyrosine-derivative and a metabolite differing from 4-EPS by the methyl substitution in the phenyl ring instead of ethyl, has also been directly associated with neurodevelopmental disorders. Social behavior impairments were triggered by daily gavages of vehicle but not with antibiotics and a fecal microbiota transplant from vehicle-treated mice to recipient mice transferred the behavioral traits.^16^ The recipients also displayed decreased medial prefrontal cortex myelination and high levels of circulating *p*-cresol.

Interestingly, some microbial products may also protect from ASD behavioral features as reported by Sharon et al.^42^ in GF mice offspring colonized by human ASD microbiota. Compared to mice with microbiota from normally developing donors, the ASD mice had significantly lower levels of 5-aminovaleric acid (5-AVA) and taurine, products of amino acid metabolism by the microbiota. Moreover, administration of these metabolites during the prenatal period or before reaching postnatal age of 4 weeks ameliorated ASD-like behavior in the offspring. Maternal obesity-induced cognitive and social deficits in the offspring were also reversed by post-weaning supplementation of short-chain fatty acids (SCFAs) acetic and propionic acid together with improvements in synaptic ultrastructure and microglial maturation in the hippocampus.^45^ Microbiota signaling during a critical development period was also required for fear extinction learning and related neuronal plasticity in mice.^38^ Moreover, multi-site metabolomics revealed four phenolic derivatives and sulfates: phenyl sulfate, pyrocatechol sulfate, 3-(3-sulfooxyphenyl)propanoic acid and indoxyl sulfate, as potential microbiota-derived signaling molecules in the context of fear learning and behavior.

In a recent work, Vuong et al.^28^ demonstrated that maternal microbiota has a crucial role in fetal thalamocortical axonogenesis, the axonal branching connecting thalamus to the cortical areas of the brain. Subsequently, absence or depletion of maternal microbiota impaired the neurobehavioral responses of the adult offspring. Colonization by *Clostridia*-dominant bacteria was sufficient to restore fetal axonogenesis, and the effect was mediated through selected bacteria-derived metabolites. TMAO, 5-AVA, 5-aminovaleric acid betaine (5-AVAB), imidazolepropionic acid, and hippuric acid were found to promote axonogenesis both *in vitro* and *in vivo*, in the absence of live bacteria.^28^ Although the microbiota-dependency and maternal translocation of the named metabolites to the fetus have been reported in several works,^67,130–132^ their contribution to host neurodevelopment is a novel finding likely to spark intensive further research. Additionally, the neurogenic properties of metabolites may not be limited to early life since indole, a tryptophan metabolite, was found to increase neurogenesis in the hippocampus of adult mice.^36^ The effect mediated by aryl hydrocarbon receptor (AhR) was specific to indole because another AhR ligand and also a tryptophan-derivative, kynurenine, did not induce any changes in neurogenesis *ex vivo*.

Studies on the relationship between human neurodevelopment and gut microbiota metabolites rely on limited number of cross-sectional and associative works done mainly in children with ASD. These children display deviations in a range of sulfates when compared to normally developing children. For instance, the elevation of 4-EPS was observed in plasma^41^ in line with earlier findings of the increase in urinary and fecal *p*-cresol sulfate^43,44^ in children with ASD. De Angelis et al.^43^ also noted elevations in the fecal indole levels among the ASD group. Results on differences between fecal SCFA concentrations, fermentation products of dietary fiber, in children diagnosed with ASD compared to normally developing children have been inconclusive as increased,^46^ decreased,^43,47,133^ and comparable^134,135^ concentrations have been reported.

Human studies initiated at the early stages of life with long follow-up times and application of multi-omics approaches are essential to understand how the host–microbe interactions influence neurodevelopment. Efforts such as the first pilot studies employing fecal microbiota transplantation in children^136^ and infants^137^ open new possibilities to study whether there are long-term effects on neurodevelopment or behavior in early-life interventions targeting the gut microbiota composition and functionality.

### Neurotransmission

In addition to the production of neurotransmitters, gut microbiota, and its metabolites can influence host’s central metabolism of neuroactive compounds.^130^ The most robust examples of gut bacteria-derived neurotransmitters are aromatic amino acid derivatives dopamine and norepinephrine^138^ and glutamate derivative γ-aminobutyric acid (GABA).^14^ In the gut lumen, microbiota significantly contributes via the activity of β-glucuronidase to the levels of free dopamine and norepinephrine.^7^ However, this may not directly translate into increased brain levels of dopamine as GF mice had higher concentration of dopamine in cerebrum compared to conventionalized mice.^67^ Also, inconsistent tissue-specific variation has been observed in GABA levels as conventionalized mice exhibit substantially higher levels of GABA within the colon and the blood but not in the brain in relation to their GF counterparts.^67,139^

Kynurenic acid, a tryptophan metabolite, serves as a modulator of extracellular glutamate as it reduces glutamate levels that participate in the glutamatergic signaling in the hippocampus.^59,60^ Consequently, increasing glutamate levels through limiting hippocampal kynurenic supply enhanced cognitive abilities and memory in rats and mice.^59–61^ Deviations in the CNS and peripheral glutamate-glutamine-GABA metabolism were depicted by elevated glutamine and GABA and decreased glutamate in hippocampus while serum GABA remained unchanged in mice after fecal microbiota transplantation from schizophrenic patients.^140^ However, a 4-week treatment with *Lactobacillus rhamnosus* increased glutamate, GABA and N-acetyl aspartate concentrations in mice brains, returning to baseline after 4-weeks of treatment cessation.^55^ *L. rhamnosus* treatment was also shown to alter brain GABA receptor expression in a regionally dependent manner and reduce stress-induced corticosterone and anxiety- and depression-like behavior in mice.^5^ These effects occurred through vagal communication as vagotomy prevented such changes suggesting a peripheral, gut-centered route to modulate brain functions and behavior.

Over 90% of serotonin in the body is produced by the enterochromaffin cells of the gut synthesizing serotonin from tryptophan, a process where gut commensals have an important regulatory role.^8,9^ Tyramine, deoxycholic acid, and 4-aminobenzoic acid were found to stimulate serotonin synthesis both *in vitro* and *in vivo*.^9^ Moreover, microbiota-associated metabolites like norepinephrine, indole, indole-3-aldehyde, isovaleric acid, butyric acid, and isobutyric acid stimulate enterochromaffin cells to release serotonin, which then engages with serotonin-receptor-expressing nerve fibers.^52,57^ In addition, some gut bacteria can produce serotonin but the physiological significance of this is currently unknown.^68,141^

The presence of pipecolic acid in the CNS can be partially derived from the microbiota^130^ and has been associated with GABA signaling and release.^142,143^ Another metabolite with a plausible connection to GABA expression in the brain, hippocampus and frontal cortex more precisely, is lactate.^20,65^ Lactate can also induce brain-derived neurotrophic factor (BDNF) expression in the hippocampus through the upregulation of Sirtuin1 deacetylase.^66^ Together, these studies show that lactate affects neural plasticity and has a beneficial effect on learning and memory in mice. SCFAs, particularly butyric acid, may also have additional regulatory effects on the production of neurotrophic factors, such as BDNF, signal the brain via vagal nerve and induce biosynthesis of neurotransmitters in the CNS.^21^ Individual administration of SCFAs butyric, acetic, and propionic acid ameliorated stress-responsivity, anxiety- and depressive-like behavior in mice concomitant with downregulation of several hypothalamic genes involved in stress signaling.^144^ Metabolites may also have negative neuromodulatory activities: administration of *p*-cresol has been observed to modulate oxytocinergic and opioidergic systems, dopamine turnover and receptor activity in specific brain regions while eliciting negative traits in social and anxiety-like behaviors in mice or rats.^48–50^ Such findings may partially explain the harmful effects of *p*-cresol on neurodevelopment.

Selected gut microbial species can also produce neurotransmitter receptor agonists or induce local synthesis of neuroactive mediators. Phenylethylamine is a full dopamine receptor agonist produced by *Morganella morganii* strains and capable of crossing the BBB.^15^ The previously described 5-AVA and taurine may also act as weak GABA_A_ receptor agonists.^42^ Tryptamine is found in low concentrations in the brain, produced by selected bacterial strains of the *Clostridium sporogenes* and *Ruminococcus gnavus* species^145^ and acts through epithelial serotonin-receptor (5-HT_4_), a G-protein coupled receptor, increasing colonic secretion and gut transit time without affecting the colonic serotonin excretion.^71^ Indole-derivatives accumulate in the brain;^55,146^ overproduction of indole, whether acute or chronic, triggered anxiety-like behavior and stimulated vagal afferent fibers in the intestinal mucosa in rats.^55^ However, in mice, the chronic overproduction of indole had adverse effects on behavior only when mice were subjected to chronic stress.^146^ Controversially, increased indoles, especially indoleacetic acid, were linked with reduced anxiety-like behavior in mice receiving fecal microbiota transplantation from patients with irritable bowel syndrome and subsequently treated by probiotic strain *Saccharomyces boulardii*.^147^ Indole also promotes short-term glucagon-like-peptide-1 release from colonic L cells,^56^ while SCFAs induce the secretion of both glucagon-like-peptide-1 and peptide YY from the enteroendocrine cells and leptin from adipocytes, all hormones participating in the food intake regulation signaling in the central and peripheral nervous system.^10−13^

These observations described above suggest that neurotransmitters, neurotransmitter receptor agonists and other neuroactive mediators produced by the gut microbiota signal the brain via the peripheral nervous system and can alter the synthesis pathways of neurotransmitters in the brain. While the findings from clinical studies are associative and focus on plasma metabolites, they support the preclinical results, to some extent. Decreased circulating levels of serotonin are a classical sign of related brain disorders like depression and alcohol use disorder.^69,148,149^ For example, reduced circulating serotonin levels precede future diagnosis of alcohol use-related diseases even when baseline alcohol drinking is accounted for.^70^ However, the regulation of circulating serotonin levels is multifaceted and influenced by external factors, such as diet.^148,150^ The situation of serotonin system in the brain is even more complex, since serotonin is an important modulator of neurotransmission and therefore the brain levels are controlled differently to the levels in the blood.^148,149^ The tryptophan metabolism, especially increased catabolism of tryptophan through the kynurenine pathway, has been linked with depression and ASD in humans.^151^ Arnoriaga-Rodríguez et al.^58^ showed that while tyrosine and tryptophan correlated positively with memory scores, the associations between their catabolites and memory scores were only evident in the obese but not in lean subjects and bacterial functions related to tryptophan and phenylalanine metabolism were negatively correlated with performance in memory tests. Cognitive functions such as learning were also negatively correlated with plasma kynurenine/tryptophan ratio in female subjects with medically diagnosed depression.^62^ A meta-analysis of the peripheral blood metabolites in medically diagnosed patients showed consistent reduction of tryptophan and kynurenic acid relative to healthy controls.^63^ Very recent data associated urinary excretion of indoxyl sulfate, the major final metabolite of indole, to recurrent depressive symptoms in female patients.^89^ Administration of the so-called psychobiotics – probiotic strains that could benefit mental health – reduced plasma kynurenine level while improving cognitive functions^64^ and increased gene expressions related to the tryptophan-serotonin metabolism in stressed adults.^152^ Valles-Colomer et al.^141^ found a positive correlation between the mental quality of life score and the microbial pathway synthesizing a dopamine metabolite 3, 4-dihydroxyphenylacetic acid. However, 3, 4-dihydroxyphenylacetic acid has also been proposed as a biomarker of neurodegenerative disorders, mainly Alzheimer’s disease.^153^

### Blood–brain barrier integrity

The BBB is a semipermeable diffusion barrier separating circulating blood and brain regions, apart from the circumventricular organs.^154^ Together with astrocytes, pericytes, microglia, neurons, and extracellular matrix, the brain microvascular endothelial cells connected by tight junction proteins form a dynamic cellular system maintaining brain homeostasis. In its neuroprotective role, the barrier restricts pathogens, peripheral immune factors, and large molecules from entering the brain, also limiting the passage of gut metabolites to the CNS. Gut microbiota is vital for the normal barrier function during pre- and postnatal periods as BBB permeability was increased in GF compared to specific pathogen-free mice.^73^ Moreover, colonizing GF mice with butyric acid-producing *C. tyrobutyricum* or acetic and propionic acid-producing *Bacteroides thetaiomicron* or oral administration of sodium butyrate restored the BBB permeability to the status seen in the controls. The administration of sodium butyrate increased the expression of occludin in the frontal cortex and hippocampus and increased brain histone deacetylation, which was also detected in *C. tyrobutyricum* inoculated GF mice. Accordingly, in a mice model of traumatic brain injury characterized by severe BBB disruption, a single intraperitoneal injection of sodium butyrate reproduced the partially alleviating effects on BBB integrity through increased expression of zonula occludens-1 (ZO) protein and occludin.^74^

Addition of a physiologically relevant dose of propionic acid to an *in vitro* model of human BBB showed the modulatory effect being independent of tight junction protein expression.^78^ Instead, propionate promoted BBB integrity by mitigating oxidative and pro-inflammatory pathways and by reducing the expression of a specific efflux transporter low-density lipoprotein receptor-related protein 1. High affinity monocarboxylate transporters facilitate the translocation of SCFAs and are abundantly expressed on the endothelial cells, neurons, and astrocytes.^21^ Furthermore, SCFA receptor FFAR3 is widely expressed in the sympathetic nervous system and has been found in rat – but not in mice – brain tissue. However, contrary to the verdicts from *in vitro* and rat studies, the uptake of SCFAs into the CNS seems to be nominal in humans as peripheral levels are in µmolar range compared to the picomolar range observed in the brain tissue.^21^ This implies that the effects of SCFAs may rather result from peripheral signaling instead of direct uptake to the brain as seen in animal models.

Aside from the SCFAs, secondary bile acids deoxycholic acid and ursodeoxycholic acid may modulate BBB integrity. Injection of deoxycholic acid in rats increased BBB permeability demonstrated by colorimetric assay and albumin immunoreactivity.^75^ The mechanism was mediated by disruption of the tight junction proteins occludin and ZO-1 and ZO-2. On contrary, in an *in vitro* model mimicking the effect of severe hyperbilirubinemia on BBB, ursodeoxycholic acid partially protected endothelial cells from apoptosis and partially restored the barrier integrity.^80^

Lastly, recent data sustain that trimethylamine (TMA), a metabolite derived from dietary choline, betaine, and L-carnitine had a dose-dependent detrimental impact on the barrier integrity in an *in vitro* BBB model by impairing the actin cytoskeleton and inducing metabolic stress.^27^ Interestingly, the oxidized form of TMA, TMAO, improved the barrier integrity both *in vitro* and *in vivo* when physiologically relevant doses were used. Systemic administration of TMAO to wild-type male mice protected from lipopolysaccharide-induced damages to the BBB function and prevented the loss of performance in the working memory test. Similarly, a host–gut co-metabolite *p*-cresol glucuronide improved BBB integrity *in vivo* and prevented lipopolysaccharide-induced permeabilization of the BBB *in vitro* by acting as a Toll-like receptor 4 antagonist.^72^

**Figure 2.**
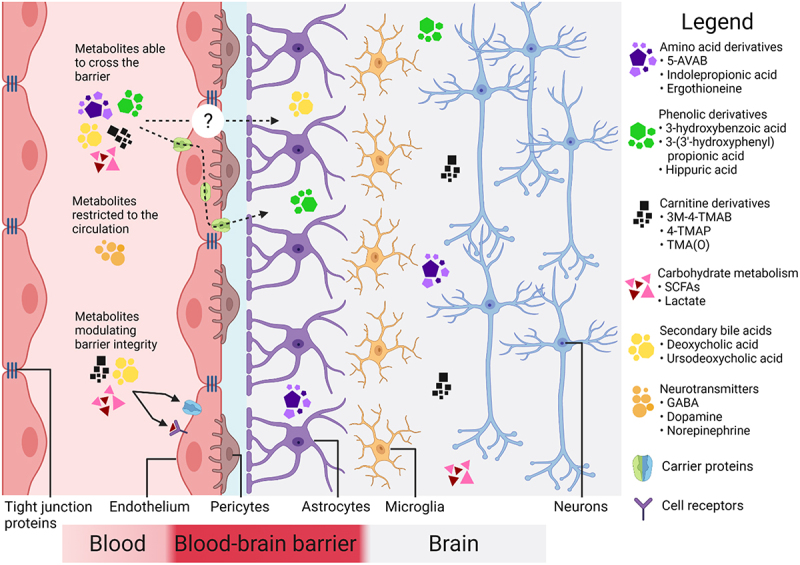
**The integrity and selectivity of the blood**–**brain barrier in terms of microbial metabolites.**

Selectivity of the BBB restricts the passage of most metabolites to the brain although transporters, diffusion, or transcytosis could facilitate crossing of the BBB of certain compounds (Figure 2) corroborating the evidence from GF mice.^28,54,131^ Nevertheless, microbial metabolites such as 5-AVAB, TMAO, *p*-cresol sulfate, and hippuric acid among others have been found in the human brain.^54^ In addition to translocation to the brain, the metabolites could alter barrier function to some extent but the physiological significance of this in humans is yet to be determined. In Alzheimer’s disease patients the increase in microbially produced deoxycholic acid and its taurine or glycine conjugated forms in the serum had a strong association with cognitive impairment^76^ and cerebrospinal fluid t-tau aggregation.^77^ A gradual increase in the plasma lithocholic acid along the disease progression over follow up time of 9 years has also been recorded.^155^ In the brain, the concentrations of taurolithocholic, 3-dehydrochenodeoxycholic, and ursodeoxycholic acid were significantly higher in relation to controls.^81^ The findings from genome-scale reconstructions suggested that the potential to synthetize these molecules is limited to a small number of bacteria species^156^ that could be the focus of future studies to scrutinize the role of bacterial bile acid transformation and BBB integrity in neurodegenerative diseases.

The blood–brain barrier restrains the passage of most microbially produced molecules from the circulation to the central nervous system. Only certain amino acids, carnitine, and phenolic derivatives together with secondary bile acids and products of carbohydrate metabolism may cross the blood–brain barrier. Carrier proteins such as monocarboxylate transporters, receptor-mediated or adsorptive transcytosis facilitate the metabolite’s translocation. Instead of crossing the blood–brain barrier, metabolites may alter the blood–brain barrier integrity resulting into increased permeability and translocation. Abbreviations: 5-AVAB, 5-aminovaleric acid betaine; GABA, ɣ-aminobutyric acid; SCFA, short-chain fatty acid; 3M-4-TMAB, 3-methyl-4-(trimethylammonio)butanoate; 4-TMAP, 4-(trimethylammonio)pentanoate; TMA(O), trimethylamine (-*N*-oxide). (Figure created with Biorender.com)

### Neuroinflammation

The inflammatory responses of the CNS to infection, non-sterile inflammation (brain lesion, traumatic brain injury, etc.), pathological conditions, obesity/metabolic diseases (meta-inflammation), and aging (inflammaging) are characterized by the production of pro-inflammatory cytokines, reactive oxygen species, and chemokines by invading immune cells and/or resident brain cells.^157,158^ However, depending on the magnitude and extent of neuroinflammation, and the phagocytic capacity of microglia, the resident brain macrophages, the outcomes may support brain return to homeostasis or elicit neuropathological processes, such as the one associated with neurodegenerative diseases and traumatic injuries.^159^

Studies in GF mice have indicated that microbiota has an integral role in the modulation of host immune development, both locally in the intestine and in the CNS.^83,160–162^ Bacterial components are recognized by the host immune system and this constant crosstalk maintains the immune homeostasis and educates the system to discriminate pathogens from symbionts. Nevertheless, bacterial metabolites also contribute to this crosstalk and can modulate inflammation, including in the brain.^162^ For example, TMAO administration exacerbated postoperative cognitive dysfunction in rodents, possibly through its effect on increased microglia activation and neuroinflammation.^101,102^ In aged mice, the microbiota-related TMAO and *N*^6^-carboxymethyllysine were upregulated in the serum and brain tissue, but only *N*^6^-carboxymethyllysine induced microglial dysfunction by interfering with mitochondrial function and increasing oxidative stress.^92^ Contrary to aged mice, such effects were evident in young mice only after intraperitoneal, but not oral administration. The assessment of gut permeability revealed age-dependent changes in microbiota composition facilitating the disruption of gut barrier properties and concomitant translocation to the circulation and brain.

On the other hand, malformed microglia in the absence of microbiota could be restored by administration of gut microbial metabolites, such as SCFAs that reinstated the T cell balance in the CNS.^83,95^ Such findings indicate that SCFAs can participate in the immune cell maturation and homeostasis in the CNS via peripheral signaling. Recent data further indicate that SCFAs (as a mix of butyric, propionic, and acetic acid) increase microglial recruitment and reactivity.^84^ Also, reinstating the SCFA-producing floras or administration of SCFAs inhibited hippocampal neuroinflammation and neuronal apoptosis, thus ameliorating cognitive decline and depressive-like behavior in a rat model of chronic cerebral hypoperfusion.^85^ Controversially, propionic acid alone has been linked with CNS oxidative stress, astrogliosis, hyperactivity, and social behavior abnormalities in a series of rat models of ASD.^96–100^ Interestingly, SCFAs had no effect on the α-synuclein aggregation *in vitro*, but induced aggregation in selected brain regions and promoted motor deficits in an *in vivo* genetic model of Parkinson’s disease.^86^ Correspondingly, SCFAs were found to increase amyloid beta protein (Aβ) deposition and microglia derived ApoE expression in an *in vivo* model of Alzheimer’s disease but not *in vitro*.^84^ Acetate induced GF mice’s microglia maturation and restored defects in the microglial mitochondrial metabolism but decreased microglia phagocytosis in an mice model of Alzheimer’s disease resulting in increased Aβ deposition.^82^ SCFAs could be of high interest in regulating microglia activity through the maintenance of physiological neuroimmune activity and through their polarization toward a pro-resolutive phenotype in neurodegenerative diseases.

Pro-inflammatory cytokines are well-known contributors to the pathophysiology of mood disorders through their action in the brain.^163,164^ Dihydrocaffeic acid was identified as a potential suppressor of peripheral IL-6 in a mice model of stress-induced depression.^88^ This compound with antioxidant properties is a polyphenol derivative from caffeic acid and requires the biotransformation of the gut bacteria. The mechanism behind the attenuated depression symptoms after the administration of dihydrocaffeic acid was independent of changes in the brain monoaminergic pathways. Rather, it was suggested to derive from epigenetic changes in the form of DNA methylation in genes coding IL-6 resulting in decreased pro-inflammatory cytokine expression.

Tryptophan-derived metabolites are a major group of AhR activators and the significance of AhR-signaling pathway has been recently associated with intestinal immune responses and neurological signaling.^165,166^ Tryptophan is catabolized through several pathways, including the kynurenine pathway, that produces intermediates with both pro- and anti-inflammatory properties, such as indoles, although their overall significance remains controversial.^167^ For instance, indoxyl sulfate promoted oxidative stress and inflammation in mice primary astrocyte and mixed glial cell cultures and caused dose-dependent neuronal cell death.^17^ Furthermore, single administration *in vivo* was followed by histological brain alterations and increase in inflammatory markers in mice. In rats, chronic indoxyl sulfate treatment modeling patients with chronic kidney disease resulted in behavioral alterations and impairments in spatial memory and locomotor activity.^90^ Controversially, indoxyl sulfate, indolepropionic acid, and indole-3-aldehyde modulated astrocyte activation and suppressed CNS inflammation both *in vitro* and *in vivo* in an experimental autoimmune encephalomyelitis mouse model of multiple sclerosis.^32,91^ Furthermore, AhR was also activated by indoxyl sulfate in human astrocytes followed by a decrease in proinflammatory gene expression. Samples from individuals with multiple sclerosis suggested that AhR-dependent regulation was impaired together with reduced number of tryptophan-derived activators.^32^ Such contradicting results may spur from species- or disease-related differences, intensity, and duration of indoxyl sulfate administration or currently unspecified agonistic properties. Recently, urolithin A, a gut-derived metabolite of ellagic acid, has been shown to reduce neuroinflammatory processes and microglia activation, and alleviate symptoms of experimental multiple sclerosis, through AhR.^106,107^ In a mouse model of stroke, urolithin A reduced neuroinflammation and neuronal loss limiting the deficits in neurological functions.^108^

A number of preclinical works suggest that SCFAs are imperative bacterial metabolites in terms of immune modulation and neuroinflammation but with their limited translocation to the brain, the suppression of neuroinflammation is likely transmitted via peripheral signaling.^21^ Moreover, the detrimental effects of intracerebroventricular propionic acid imply that within the CNS, SCFAs might aggravate inflammatory responses.^96–99^ However, the number of clinical studies with SCFAs is limited and results on systemic inflammation markers are inconclusive.^87,168^ Bacterial metabolites that function as AhR agonists can reach the CNS and regulate inflammation, but the overall outcome is dictated by factors, such as the health status of the host and the balance between pro- and anti-inflammatory agonists.^165^ For instance, Sankowski et al.^51^ demonstrated in a pilot study that the cerebrospinal fluid/plasma ratios of indoxyl sulfate, TMAO, and *p*-cresol sulfate were 4–8 times higher in Parkinson’s disease patients than in healthy controls and their concentration in the cerebrospinal fluid was linked to a more advanced disease stage in the absence of decreased kidney function. In addition to Parkinson’s disease, TMAO-related microbial genetic pathways have been associated with Alzheimer’s disease^153^ and elevated TMAO in the cerebrospinal fluid has been recorded in patients with Alzheimer’s disease,^79^ in the plasma of patients with post-stroke cognitive impairment^103^ in addition to inverse correlation to cognitive performance^102^ in middle-aged and older humans. However, recent contradicting evidence did not support causality between TMAO and Alzheimer’s disease^104^ accompanied with findings of lower plasma TMAO in Parkinson’s patients^105^ compared to controls. Finally, the advanced glycation end product *N^6^*-carboxymethyllysine has been identified in brain tissue and associated with oxidative stress in elderly and patients with Alzheimer’s disease or diabetes.^93,94^

### Neuronal energy metabolism

The neurons, principal component of the nervous tissue use up to 80% of the brain’s energy production to maintain the excitability of the synapses.^169^ Brain cells rely mainly on glucose, although ketone bodies and lactate can be utilized flexibly to support the energy demand of the neuronal network. Astrocytes can store glucose as glycogen or metabolize it via glycolysis yielding lactate to be oxidized in neurons and this astrocyte-neuron glycogenolytic lactate production and transporting is crucial for long-term memory formation and subsequent synaptic plasticity that is not sustained by glucose delivery alone.^114^ Peripherally supplied lactate can also reach the CNS since bacteria-produced lactate was identified as the source of memory enhancements in mice.^20^ The lactate production in astrocytes is also entangled with the glutamine-glutamate cycle, possibly linking the observed effects to the downstream production of neurotransmitters glutamate and/or GABA, both participating in learning and memory functions.^170,171^ Nevertheless, lactate itself augments neural activity as the primary energy source and the lactate receptor GPR81 is enriched in regions of cerebral neocortex, hippocampus, and the BBB highlighting its several functions in neural processes.^172,173^

Fecal microbiota transplantation from subjects with alcohol use disorders to recipient mice resulted in enrichment of ethanol-producing bacterial species, reduction in lipolysis, and the supply of ketone bodies in the circulation.^113^ Specifically, depletion of β-hydroxybutyrate was associated with increased inflammatory responses and decreased markers of myelination and social behavior. The latter findings were also replicated in a cohort of subjects with alcohol use disorder.^113^ Moreover, ketogenic meals or administration of β-hydroxybutyrate has been shown to improve memory or cognitive performance in elderly or type 2 diabetic subjects.^174,175^ Also, the GABAergic neurons can utilize β-hydroxybutyrate to produce neurotransmitters GABA and glutamate,^176^ which are altered in the postmortem brains of persons with history of heavy alcohol use.^54^

Recently, intermittent fasting was shown to improve cognitive function via gut–brain axis in *db*/*db* mice and simultaneously modulate brain energy metabolism in hippocampus.^110^ Fasting significantly increased the plasma levels of indolepropionic acid and tauroursodeoxycholic acid (TUDCA) and fecal levels of SCFAs. Individual administrations of indolepropionic acid or TUDCA or a mixture of SCFAs acetic, propionic and butyric acid were able to reproduce the effects of fasting in cognition, hippocampal mitochondrial biogenesis, and energy metabolism-related gene expression. Previously, incubating mice brain mitochondrial preparations with indolepropionic acid supported mitochondrial function by increasing membrane potential and inhibited generation of free radicals.^177^ Indolepropionic acid has also been shown to increase mitochondrial respiratory rates in amyloid precursor protein expressing neuroblastoma cell cultures.^115^ On the contrary, butyric or propionic acid treatments have been associated with disturbed mitochondrial fatty acid metabolism in a rat model of ASD^111^ but butyric acid alone improved mitochondrial function in lymphoblastoid cell line derived from ASD boys.^112^ Recently, Hulme et al. identified two novel microbial-derived structural analogues of carnitine, 3-methyl-4-(trimethylammonio)butanoate (3M-4-TMAB), and 4-(trimethylammonio)pentanoate (4-TMAP), that were found to colocalize with carnitine in brain white matter.^109^ The fatty acid oxidation in mitochondria is in part mediated by carnitine and in subsequent *in vitro* cell models of murine white matter the metabolites were observed to inhibit the rate of fatty acid oxidation, thus interfering with mitochondrial function. An isomer of the compounds, gut microbiota-associated compound 5-AVAB, has also been shown to limit fatty acid oxidation in mouse cardiomyocytes by interfering with carnitine transportation into the cell.^178^

Impairments in mitochondrial functions are considered as independent drivers of cognitive decline observed in the aging brain, neurodegenerative disorders, and neurodegeneration caused by external exposures like alcohol.^179^ Also, mitochondrial dysfunction has been associated with ASD.^180^ Thus, approaches targeting and supporting brain bioenergetics should be evaluated in the future. Findings connecting microbial metabolites to brain bioenergetics are preliminary but show that certain compounds are incorporated in the neuronal energy metabolism. However, the overall impact of many of these metabolites on mitochondrial output has not been investigated in humans making their contribution to brain health uncertain.

### Neuroprotection

Actions resulting in preservation or recovery of the neuronal cells, their structural network or function are defined as neuroprotective. Hence, metabolites of the gut microbiota reducing oxidative stress or aggregation of neurotoxic proteins can be considered as neuroprotective agents. In this context, metabolites that reduce inflammation or promote neurodevelopment or neurotransmission are also neuroprotective.

The number of phytochemicals with suggested neuroprotective effects is considerable and here we cover only few examples of gut microbial metabolites as there are other focused articles available (for reviews, see refs.^181–183^). For instance, ferulic acid is a phytochemical associated to dietary fibers present in many plant products, released from the fiber matrix, and further metabolized by the gut microbes.^184^ Ferulic acid holds neuroprotective effects by limiting neuronal cell death and improves memory deficits in a mouse model of cerebral ischemia and reperfusion injury.^18^ In a mouse model of corticosterone administration-induced depression, ferulic acid ameliorated depression-like behavior and oxidative stress.^119^ A microbiota-borne metabolite and a downstream product of ferulic acid, dihydroferulic acid, also exhibits neuroprotective antioxidative properties *in vitro*.^19^

Phytochemicals have also shown potential neuroprotective properties in cell and murine models of Alzheimer’s disease where the aggregation of neurotoxic Aβ fragments were inhibited in the presence of polyphenol metabolites. For example, ferulic acid reduced hyperactivity and Alzheimer’s disease-related pathological changes in the brain while improving spatial working and reference memory in a murine model of cerebral amyloidosis.^120^ Metabolites of flavonoids and phenolic acids, 3-hydroxybenzoic acid and 3-(3′-hydroxyphenyl)propionic acid, were shown to accumulate in mice brain at µM concentration following administration of grape seed polyphenol extract or red wine and inhibit the formation of Aβ aggregates *in vitro*.^116^ In preclinical models of neurodegenerative diseases called synucleinopathies, vitamins K (phylloquinone, menaquinone, and menadione) produced by the microbiota did inhibit the aggregation of the protein α-synuclein within the neurons *in vitro*.^122^ Ergothioneine is a histidine derivative produced by gut bacteria that is translocated to the brain and protects from oxidative stress as well as Aβ-induced cellular damage *in vitro* and *in vivo*.^117,118^ Finally, TUDCA has been shown to reduce neuronal apoptosis in several other preclinical models of neurodegenerative disease such as Huntington’s, Alzheimer’s and Parkinson’s diseases and acute ischemia and interfere with Aβ production^123,185^ while addition of indolepropionic acid prevented oxidative stress and neuron apoptosis in neuroblastoma cell cultures exposed to Aβ.^121^

A range of gut-derived metabolites exert neuroprotective properties in *in vitro* and *in vivo* preclinical models, but convincing clinical studies are lacking. For example, administration of plant or fruit extracts to humans has improved cognitive functions and mood in various feeding trials, but these studies did not explore the effect on the metabolome.^183^ Nevertheless, the accumulating number of reports showing that amino acid- and polyphenol-derivatives interfere with the formation of neurotoxic proteins, oxidative, and inflammatory cascades warrant for further clinical studies targeting patients with neurodegenerative diseases.

## Enteric nervous and immune systems as pathways of communication

### Enteric nervous system

**Figure 3.**
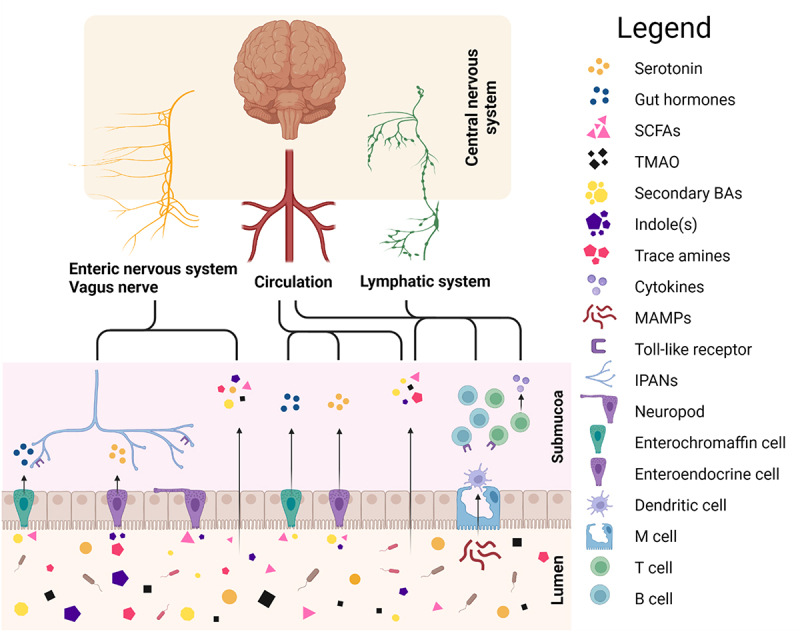
Communication routes between the gut and brain.

Without ever reaching the circulation and brain, the microbial metabolites can begin communication toward the brain through the gastrointestinal sites of nervous and immune systems (Figure 3). The enteric nervous system is a neuronal network of sensory neurons, motor neurons, interneurons, and supporting cells such as enteric glial cells embedded in the intestinal wall throughout the gastrointestinal tract.^186^ As the endings of the intrinsic primary afferent neurons are in the submucosa, the enteric nervous system is the first neuronal interface that could be directly or indirectly activated by the microbial metabolites.^187^ While it can operate functions such as local motility and secretion autonomously, this component of autonomic nervous system is connected to the CNS via sympathetic and parasympathetic pathways forming a loop of gut–brain–gut signaling. The sensory information from the gut to the brain is carried by primary afferent neurons of the vagal and spinal branches of which the former have been suggested as a key transmitter of gut microbiota stimulus. Indeed, the vagus nerve is composed of up to 90% of afferent fibers and depending on the location and type of the afferent fiber, they are stimulated by neurotransmitters, gut microbial metabolites, or gut hormones.^188^ Consequently, it has been shown that the vagus nerve is indirectly activated by the gut microbiota-associated metabolites such as serotonin,^57^ SCFAs,^4^ and indole.^55^ Moreover, the vagal pathways are also linked with the hypothalamic–pituitary–adrenal axis, which is a major regulator of stress responses. Coupled with the findings that vagotomy affects brain functions^5^ and vagus nerve stimulation modulates mood and behavior,^188^ the enteric nervous system–vagus–brain pathway is likely one of the main mechanisms of gut–brain axis communication.

Schematic representation of various potential communication pathways connecting the gut to the brain. Gut microbiota-derived metabolites can interact with the enteral nervous system directly or activate it indirectly via colonic enterochromaffin or L cells’ enteroendocrine function. Alternatively, metabolites or gut hormones can translocate to the circulation or lymphatic system and eventually reach the brain. Similarly, inflammatory mediators and immune cells activated by microbiota associated molecular profiles can translocate to the brain via circulation or the lymphatic system. Abbreviations: BA, bile acid; IPAN, intrinsic primary afferent neuron; MAMP, microbiota-associated molecular profile; SCFA, short-chain fatty acid; TMAO, trimethylamine-*N*-oxide. (Figure created with Biorender.com)

Other forms of indirect enteric neuron activation are the enteroendocrine signaling by colonic L cells or enterochromaffin cells. The colonic enterochromaffin cells produce the majority of the circulating serotonin in the human blood.^189^ As discussed previously, several bacterial metabolites have been shown to influence the enterochromaffin cells’ serotonin synthesis.^9^ Enteric neurons in the submucosa are in close proximity of the translocated serotonin from the lumen and express serotonin receptors. In addition to neuronal stimulation, serotonin might have a role in the development, maturation, and protection of the enteric nervous system.^190^ Similar to enterochromaffin cells, colonic L cells are activated by microbial metabolites, such as SCFAs and secondary bile acids among others.^188^ The L cells produce bioactive peptides such as GLP-1 and PYY both known for their local effects on gut motility and secretion, and central effects on food intake and feeding behavior.^191–193^ The hormones released by L cells have multiple effectors, including enteric neurons and vagus nerve, that carry the signal toward CNS.^194^ However, recently Bohórquez et al.^195^ uncovered a neuroepithelial circuit connecting the enteroendocrine cell to sensory neurons, together referred as neuropods. This suggests that among the endocrine signaling, the enteroendocrine cells could form physical connections with the neurons as a conduit for sensory transmission activated by microbial metabolites.

In theory, the microbiota’s production of neurotransmitters, for example, histamine, serotonin, or GABA, could directly engage with the enteric neurons in a paracrine manner.^194^ However, it is questionable whether microbiota-derived neurotransmitters could reach enteric neurons in meaningful concentrations. The enteric neurons also express pattern recognition receptors, including the Toll-like receptors, that are activated by microbial molecules.^194^ The significance of the Toll-like receptors in the physiology of the enteric nervous system has been shown in mice, where the lack of these receptors reduced gut motility and produced neuronal defects, both reversed by the administration of Toll-like receptor agonist to naïve animals.^196^ Moreover, in the absence of gut microbiota, the maturation and function of enteric nervous system is compromised underlining the significance of microbial cues in normal gut physiology.^194^

### Immune system

The cells of innate and adaptive immune systems in the gastrointestinal tract are the first responders to immune challenges produced by the gut microbiota.^197^ Reports from GF animals have suggested that the microbiota–host immunity crosstalk, especially in the early life, is imperative for the development and modulation of the immune homeostasis.^6,160^ The innate immune system detects microbial components through pattern-recognizing receptors, which are activated by microbe-associated molecular patterns.^197^ As with enteric neurons, Toll-like receptors are central pattern-recognizing receptors detecting microbe-associated molecules such as lipopolysaccharides, glycolipids, or lipopeptides. Activation of the innate immune response results into a signaling cascade, increasing the production of cytokines and chemokines and the recruitment of local macrophages and dendritic cells. These cells also act as antigen-presenting cells to further trigger local adaptive immune responses facilitating the migration of circulating T- and B-lymphocytes to the site of inflammation. Apparently, some gut-derived metabolites like the SCFAs may have a regulatory role in the intestinal T cell differentiation.^129,198^

As circulating lymphocytes may infiltrate peripheral tissues, similarly the cytokines or activated immune cells can be transported to the CNS and modulate the immune homeostasis.^199^ While the BBB restricts the passage of immune cells but not cytokines, the lymphatic system is an alternative route facilitating migration of these cells and bacterial components from the periphery to the CNS.^200^ The meningeal lymphatic vessels reach the CNS, exchange the cerebrospinal fluid, and drain immune cells and small compounds from the brain toward the periphery. Direct supply of microbiota-derived molecules or activated immune cells could induce a central immune response, such as the activation of microglia and T cells.^197^ This in turn leads to the production of pro-inflammatory cytokines promoting neuroinflammation. Cytokines may also deteriorate BBB permeability that predisposes to increased passage of harmful compounds or metabolites from the circulation through the BBB.^200^ Moreover, peripheral administration of pro-inflammatory cytokines has been shown to induce sickness behavior characterized by depressive-like behavior and reduced appetite in rodents.^199^ However, the range of mechanisms describing microbially mediated crosstalk between peripheral and central immune responses are widely uncharacterized.

## Microbial metabolites and the relationship between preclinical and clinical evidence

**Figure 4.**
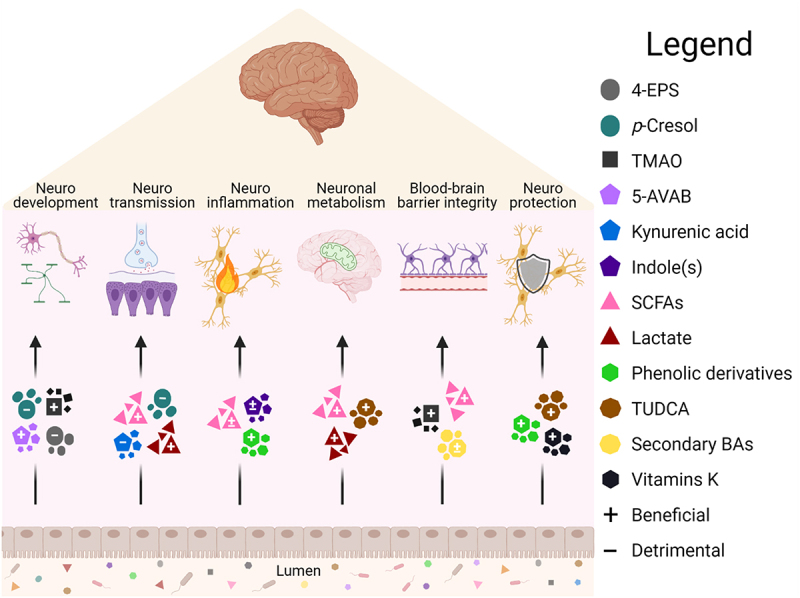
**In the intersection of the gut**–**brain axis signaling: Selected microbiota metabolites and associated neurological functions.**

A wealth of preclinical discoveries has clarified the extensive range of pathways through which microbiota-derived metabolites could drive the communication between gut and brain as illustrated in Figure 4. The human microbiome harbors a variety of microbial species capable of producing neurotransmitters, signaling molecules, or metabolizing their precursors into distinct compounds.^145,189,201^ As certain bacterium may contribute significantly to the levels of gut-derived metabolites observed in the host,^138^ it is compelling to speculate how much the presence, or absence, of such bacterium could modulate the gut–brain crosstalk and contribute to overall health status. For example, abundance of secondary bile acid-producing bacteria could result into increased circulation of deoxycholic acid and other secondary bile acids compromising the BBB integrity and resulting into increased translocation of microbial metabolites into the brain.^81^ Keeping in mind that the overall impact of these metabolites on health could depend on the genetic background or the presence of an illness, decreased liver or kidney function could exacerbate the accumulation of toxic metabolites like *p*-cresol and indoxyl sulfate that have been associated with neurodegenerative or neurodevelopmental diseases.^16,17,48–50,90^

Brain functions influenced by the gut microbiota metabolites. Certain metabolites like indoles or secondary bile acids have been shown to have both beneficial (+) and detrimental (-) effects depending on the pathway of action or disease model. Signaling pathways can be initiated in the gut via peripheral routes or after translocation to the central nervous system. Abbreviations: 4-EPS, 4-ethylphenylsulfate; 5-AVAB, 5-aminovaleric acid betaine; BA, bile acids; SCFA, short-chain fatty acid; TMAO, trimethylamine-*N*-oxide; TUDCA, tauroursodeoxycholic acid. (Figure created with Biorender.com)

**Figure 5.**
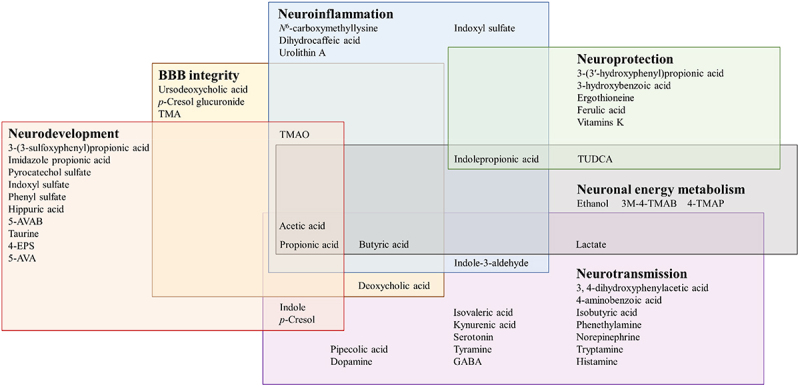
Overlapping gut microbiota associated metabolites in neurological functions.

Categorizing gut microbial metabolites based on their effect on neurological function reveals significant overlapping between metabolite classes and individual metabolites (Figure 5, Table 2). In preclinical studies, SCFAs have been implicated with all discussed neurological functions but human evidence is scarce and contradictory.^21^ Similarly, derivatives of amino acid metabolism, especially tryptophan, have been implicated in all other concepts except for neuronal energy metabolism. However, clinical findings support the notion that alterations in tryptophan-metabolism might be associated with cognitive and mental status.^58,62–64,141,151,152^ Although preclinical models display controversial impact of secondary bile acids, they have recently been associated with neurodegenerative diseases in humans.^76,77,81,155^ 4-EPS, *p*-cresol sulfate and indoxyl sulfate are sulfates that show conflicting effects on neurodevelopment, -transmission, and -inflammation *in vitro* and *in vivo* but the clinical results, although associative, favor the harmful properties depicted by the preclinical data.^41,43,44,51^ However, the findings are largely obtained in a pathological context, yielding a wealth of disease-associated metabolites. Whether such associations are disease-specific or the metabolites, or microbiota, could be utilized in managing brain-related disorders, requires extensive and decisive work. Besides, the associations might reflect the altered metabolism or microbiota composition due to the disease status and confounders rather than contribute to the pathogenesis *per se*. For example, a recent stool metagenomics study in ASD children suggested that autistic traits promote restrictive dietary preferences in turn associated with microbiome composition, rather than a direct association between the ASD and microbiome.^203^

Individual gut microbiota metabolites have been associated with single or several neurological functions involving neuronal energy metabolism, blood–brain barrier integrity, neurodevelopment, -inflammation, -transmission, and -protection. Abbreviations: 4-EPS, 4-ethylphenylsulfate; 5-AVA(B), 5-aminovaleric acid(betaine); BBB, blood–brain barrier; GABA, γ-aminobutyric acid; 3M-4-TMAB, 3-methyl-4-(trimethylammonio)butanoate; 4-TMAP, 4-(trimethylammonio)pentanoate; TMA(O), trimethylamine(-*N*-oxide); TUDCA, tauroursodeoxycholic acid.

**Table 2.** Gut microbial metabolites associated neurological functions, methods of preclinical models and presence in the human brain.

Metabolite	Neurological function	Preclinical methods	Measured from human brain^51,54,155,202^	Reference
3-(3′-hydroxyphenyl)propionic acid	Neuroprotection	*in vitro, in vivo*	Yes	^116^
3-(3-sulfooxyphenyl)propionic acid	Neurodevelopment	*in vivo*	No	^38^
3-hydroxybenzoic acid	Neuroprotection	*in vitro, in vivo*	Yes	^116^
3M-4-TMAB	Neuronal energy metabolism	*in vitro, in vivo*	No	^109^
4-aminobenzoic acid	Neurotransmission	*in vitro, in vivo*	No	^9^
4-EPS	Neurodevelopment	*in vivo*	No	^39–41^
4-TMAP	Neuronal energy metabolism	*in vitro, in vivo*	No	^109^
5-AVA	Neurodevelopment	*in vitro, in vivo*	Yes	^42^
5-AVAB	Neurodevelopment	*in vivo, ex vivo*	Yes	^28,131^
Acetic acid	Neurodevelopment Neurotransmission BBB integrity Neuroinflammation Neuronal energy metabolism	*in vitro, in vivo, ex vivo*	Yes	^45,73,83–87,110,144,163^
Butyric acid	Neurotransmission BBB integrity Neuroinflammation Neuronal energy metabolism	*in vitro, in vivo, ex vivo*	Yes	^52,73,74,83–87,110–112^
Deoxycholic acid	Neurotransmission BBB integrity	*in vitro, in vivo*	Yes	^9,75^
Dihydrocaffeic acid	Neuroinflammation	*in vitro, in vivo*	No	^88^
Dopamine	Neurotransmission	*in vivo*	Yes	^7^
Ethanol	Neuronal energy metabolism	*in vivo*	Yes	^113^
Ergothioneine	Neuroprotection	*in vitro, in vivo*	Yes	^117,118^
Ferulic acid	Neuroprotection	*in vitro, in vivo*	No	^18,119,120^
GABA	Neurotransmission	*in vitro, in vivo*	Yes	^5,14,53^
Hippuric acid	Neurodevelopment	*in vivo, ex vivo*	Yes	^28^
Histamine	Neurotransmission	*in vitro, in vivo*	Yes	^15^
Imidazole propionic acid	Neurodevelopment	*in vivo, ex vivo*	No	^28^
Indole-3-aldehyde	Neurotransmission Neuroinflammation	*in vivo, ex vivo*	No	^32,57,91^
Indole	Neurodevelopment, Neurotransmission	*in vitro, in vivo, ex vivo*	Yes	^32,36,55–57,91^
Indolepropionic acid	Neuroinflammation Neuronal energy metabolism Neuroprotection	*in vitro, in vivo, ex vivo*	No	^32,110,115,121^
Indoxyl sulfate	Neurodevelopment Neuroinflammation	*in vitro, in vivo*	Yes	^17,32,38,51,90,91^
Isobutyric acid	Neurotransmission	*in vitro, ex vivo*	Yes	^52^
Isovaleric acid	Neurotransmission	*in vitro, ex vivo*	Yes	^52^
Kynurenic acid	Neurotransmission	*in vitro*	Yes	^59–61^
Lactate	Neurotransmission Neuronal energy metabolism	*in vivo*	Yes	^20,65,114,144^
*N^6^*-carboxymethyllysine	Neuroinflammation	*in vitro, in vivo*	Yes	^92–94^
Norepinephrine	Neurotransmission	*in vitro, in vivo, ex vivo*	Yes	^7,52^
*p*-Cresol	Neurodevelopment Neurotransmission	*in vitro, in vivo*	Yes	^16,48–51^
*p*-Cresol glucuronide	BBB integrity	*in vitro, in vivo*	No	^72^
Phenethylamine	Neurotransmission	*in vivo*	Yes	^15^
Phenylsulfate	Neurodevelopment	*in vivo*	No	^38^
Pipecolic acid	Neurotransmission	*in vivo*	Yes	^67^
Propionic acid	Neurodevelopment Neurotransmission BBB integrity Neuroinflammation Neuronal energy metabolism	*in vitro, in vivo, ex vivo*	Yes	^27,45,73,83–86,95–100,111^
Pyrocatechol sulfate	Neurodevelopment	*in vivo*	No	^38^
Serotonin	Neurotransmission	*in vivo*	Yes	^9,68,141^
Taurine	Neurodevelopment	*in vivo*	Yes	^42^
TMA	BBB integrity	*in vitro*	Yes	^27^
TMAO	Neurodevelopment BBB integrity Neuroinflammation	*in vitro, in vivo, ex vivo*	Yes	^27,28,101,102^
TUDCA	Neuronal energy metabolism Neuroprotection	*in vitro, in vivo*	Yes	^110,123^
Tryptamine	Neurotransmission	*in vitro, in vivo*	Yes	^71^
Tyramine	Neurotransmission	*in vitro, in vivo*	Yes	^9^
Urolithin A	Neuroinflammation	*in vitro, in vivo, ex vivo*	No	^106–108,168^
Ursodeoxycholic acid	BBB integrity	*in vitro*	Yes	^80^
Vitamins K	Neuroprotection	*in vitro*	Yes	^122^

Abbreviations: 4-EPS, 4-ethylphenylsulfate; 5-AVA(B), 5-aminovaleric acid (betaine); BBB, blood–brain barrier; GABA, γ-aminobutyric acid; 3 M-4-TMAB, 3-methyl-4-(trimethylammonio)butanoate; 4-TMAP, 4-(trimethylammonio)pentanoate; TMA(O), trimethylamine(-*N*-oxide); TUDCA, tauroursodeoxycholic acid

In terms of the neurological functions, metabolites linked with BBB integrity, neuronal energy metabolism, or neuroprotection, the clinical evidence are extremely limited for evaluating their significance in humans. Given the value of these functions to brain homeostasis and the range of diet-derived metabolites identified, especially amino acid- and polyphenol-derived, future interventions focusing on these pathways and compounds are strongly advocated. To date, few studies using nutritional or gut microbiome modifying strategy have shown marked beneficial effect on neurological and psychiatric disorders. Even if some studies applying nutritional, pro- or prebiotic interventions showed interesting outcomes such as decreasing anxiety levels or improving cognition, the effects are widely variable between subjects and dependent on the type of diseases.^204–206^

## Conclusion and future prospects

An array of preclinical reports show that the gut microbial metabolites regulate brain functions through peripheral and local signaling by immunological, neuronal, and endocrinological pathways. Cell and animal models provide valuable mechanistic information about the metabolites in isolation but there is limited clinical evidence to support such effects in humans. Nevertheless, it is evident that nutritional, metabolic, and disease status modulate the overall impact of metabolites on health and disease. Thus, to catch the full scope of the relationship between diet, gut microbiota, the circulating metabolome and the brain, extensive profiling of nutritional habits and clinical features are fundamental. In order to gain a comprehensive view on the microbial species involved, metabolites they produce, and metabolic effect exerted thereafter, necessitates combining metagenomic, metabolomic, and metatranscriptomic approaches in well-defined populations, healthy and disease, to uncover the intricate communication within the gut–brain axis and pave the way for personalized care involving the role of gut microbiota in the context of prevention and treatment of neurological disorders.
